# ER Stress Negatively Modulates the Expression of the miR-199a/214 Cluster to Regulates Tumor Survival and Progression in Human Hepatocellular Cancer

**DOI:** 10.1371/journal.pone.0031518

**Published:** 2012-02-16

**Authors:** Quanlu Duan, Xingxu Wang, Wei Gong, Li Ni, Chen Chen, Xingxing He, Fuqiong Chen, Lei Yang, Peihua Wang, Dao Wen Wang

**Affiliations:** 1 Department of Internal Medicine and Gene Therapy Center, Huazhong University of Science and Technology, Wuhan, People's Republic of China; 2 Institute of Liver Diseases, Tongji Hospital, Tongji Medical College, Huazhong University of Science and Technology, Wuhan, People's Republic of China; University of Hong Kong, Hong Kong

## Abstract

**Background:**

Recent studies have emphasized causative links between microRNAs (miRNAs) deregulation and tumor development. In hepatocellular carcinoma (HCC), more and more miRNAs were identified as diagnostic and prognostic cancer biomarkers, as well as additional therapeutic tools. This study aimed to investigate the functional significance and regulatory mechanism of the miR-199a2/214 cluster in HCC progression.

**Methods and Findings:**

In this study, we showed that miR-214, as well as miR-199a-3p and miR-199a-5p levels were significantly reduced in the majority of examined 23 HCC tissues and HepG2 and SMMC-7721 cell lines, compared with their nontumor counterparts. To further explore the role of miR-214 in hepatocarcinogenesis, we disclosed that the ER stress-induced pro-survival factor XBP-1 is a target of miR-214 by using western blot assay and luciferase reporter assay. Re-expression of miR-214 in HCC cell lines (HepG2 and SMMC-7721) inhibited proliferation and induced apoptosis. Furthermore, ectopic expression of miR-214 dramatically suppressed the ability of HCC cells to form colonies in vitro and to develop tumors in a subcutaneous xenotransplantation model of the BALB/c athymic nude mice. Moreover, reintroduction of XBP-1s attenuated miR-214-mediated suppression of HCC cells proliferation, colony and tumor formation. To further understand the mechanism of the miR-199a/214 cluster down-expression in HCC, we found that thapsigargin (TG) and tunicamycin (TM) or hypoxia-induced unfolded protein response (UPR) suppresses the expression of the miR-199a/214 cluster in HCC cells. By promoter analysis of the miR-199a2/214 gene, we conjectured NFκB as a potential negative regulator. We further found that UPR and LPS-induced NFκB activation suppressed miR-199a2/214 transcription, and this suppression was reversed by NFκB inhibition in HCC cells.

**Conclusions:**

Our study suggest that modulation of miR-214 levels may provide a new therapeutic approach for cancer treatment and revealed that UPR may offer a new explanation for why the miR-199a/214 cluster were down-regulated in the progression in HCC.

## Introduction

MiRNAs are a new class of endogenous, non-coding RNAs 19–25 nucleotides long that mediate the repression of target transcripts by binding to complementary seed sequences at the 3′ untranslated regions (UTRs) of target mRNAs [1]. Since initial observation, more than 1400 human miRNAs have been registered in miRBase (v.17.0). Previous studies suggested dysexpression of miRNAs has been observed in various types of cancers and is also associated with the clinical outcome of cancer patients [2]. Furthermore, the abilities of miRNAs to achieve simultaneous fine tuning of numerous different target genes makes them fundamental regulators of cellular signaling and implicates them in tumor progression [3], [4]. But their specific roles and functions in the major cancers and the malignant progression of cancer have yet to be fully elucidated.

Hepatocellular carcinoma (HCC) is one of the most common cancers worldwide and among the leading causes of cancer-related death in Asia, especially in China [5]. Several miRNAs, such as miR-101 [6], miR-122 [7], [8], [9] miR-373 [10], miR-221/222 [11], [12], [13], miR-195 [14], miR-30d [15], miR-125b [16], miR-18a [17], miR-139 [18], miR-223 [19] and miR-29 [20], have already been reported to regulate HCC tumor progression and metastasis by regulating key genes such as Mcl-1, ADAM17, YAP, DDIT4, Cyclin D1, CDK6, E2F3, Galphai2, LIN28B, estrogen receptor-α, Rho-kinase 2, Stathmin 1 and Bcl-2 and so on. However, the existing data cannot fully explain the complexity of HCC.

Recently, miR-199a-3p/5p was verified to be decreased in HCC tissues, and its decrement significantly correlates with the survival of HCC patients, outlining a potential marker for predicting the prognosis of HCC patients [5], [21], [22]. It is well known that there are two genes that potentially encode pri-miR-199a, the primary precursor of hsa-mir-199a. The first gene is MIR199a1 on chromosome 19 (NCBI GeneID 406976) and the second is MIR199a2 on chromosome 1 (NCBI GeneID 406977) [23]. Interestingly, at the 3′-end of the pri-miR-199a2 transcript, there is the precursor sequence for another miRNA pair hsa-mir-214 and hsa-mir-214* [24]. miR-199a2 and miR-214 have been reported to be produced from a single intron-less transcript of Dynamin 3 opposite (Dnm3os) that is embedded in the opposite strand within an intron of Dynamin in mouse and human [23], [24]. Furthermore, the miPPR-199a2 region is shown here to be the authentic miR-199a2 promoter that produces the primary transcript harboring the miR-199a-3p, miR-199a-5p and miR-214 sequences as a cluster [25]. More and more studies documented that miR-214 is involved in human ovarian cancer, cervical cancer and melanoma tumour progression [26], [27], [28], [29]. However, the current knowledge about miR-214 expression and function in HCC is still rather unclear. In addition, the mechanisms underlying miR-199a2/214 deregulation in HCC is not yet clear.

In the present study, we showed that miR-199a-3p, miR-199a-5p and miR-214 expression was significantly reduced in HCC tissues. XBP-1 was shown to be a direct target of miR-214 by interaction with the 3′-UTR. Furthermore, the suppressive effect of miR-214 in HCC tumor formation and growth was studied *in vitro* and *in vivo*, and reintroduction of XBP-1s attenuated miR-214-mediated suppression. We further identified that NFκB activated by unfolded protein response (UPR) suppresses miR-199a2/214 transcription, and demonstrated that activation of UPR and endoplasmic reticulum (ER) stress represents an important mechanism responsible for miR-214 and miR-199a-3p/5p down-regulation in HCC development.

## Results

### miR-199a/214 is downregulated in HCC cell lines and tissues

**Figure 1:**
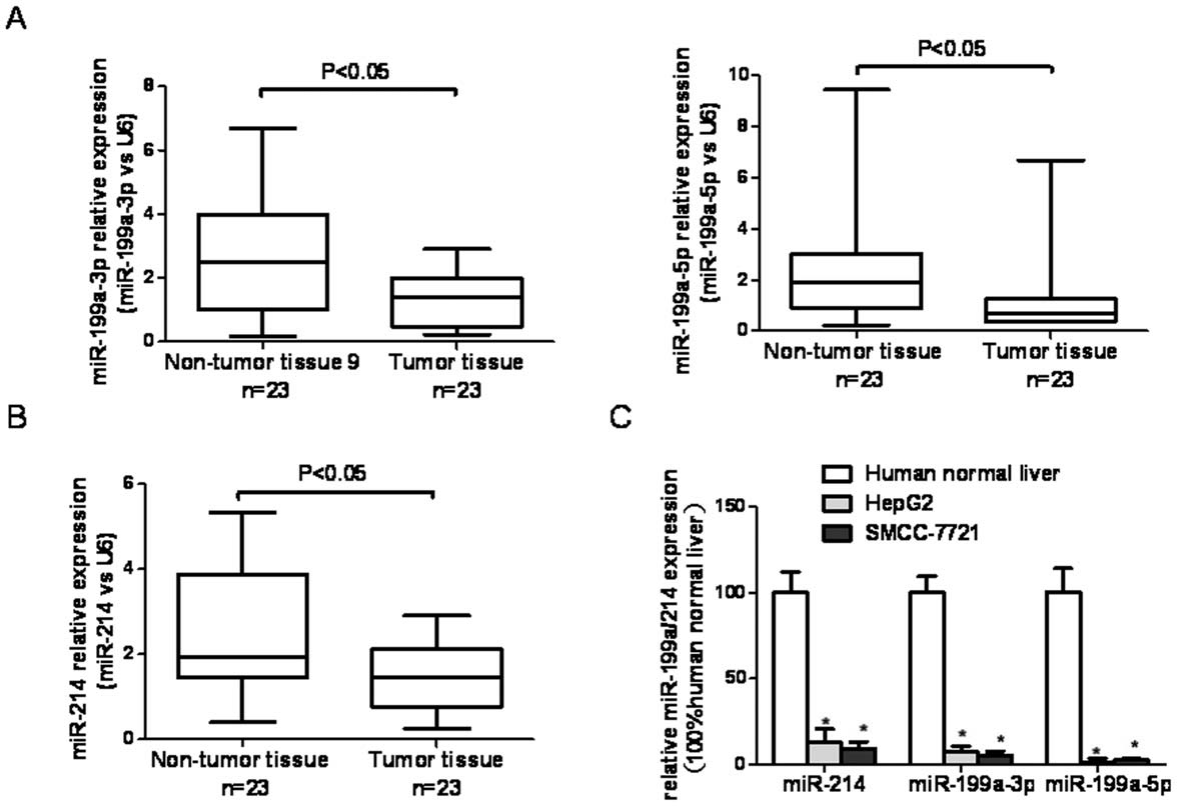
miR-199a/214 expression in HCC samples and cell lines. (**A**) Box plot graphic displays of 23 HCC and matched adjacent benign tissues grouped according to miR-199a-3p and miR-199a-5p expression. (**B**) Box plot graphic displays of 23 HCC and matched adjacent benign tissues grouped according to miR-214 expression. (**C**) miR-199a/b-3p and miR-214 expression in human normal liver, HepG2, and SMMC-7721 HCC cell lines was detected using real-time qRT-PCR. Columns, mean of three independent experiments; bars, SD; * P<0.05 vs control.

To study the role of the miR-199a/214 cluster in the HCC, levels of miR-214 and miR-199a-3p/5p were determined in 23 pairs of HCC and adjacent benign tissues using real-time PCR. Results showed that these miRNAs were all significantly down-regulated and miR-199a-3p>miR-199a-5p>miR-214 compared with adjacent nontumorous liver tissues. Decreased miR-214 expression was observed in 65% of HCC (15 of 23 cases), and consistent down-regulation of both miR-199a-3p and miR-199a-5p also were detected in as much as 73% of HCC (17 of 23 cases) (Figure 1A and B). In parallel, in HCC cell lines HepG2 and SMMC-7721, miR-199a-3p/5p and miR-214 expression was markedly decreased compared with that in human normal liver (Figure 1C).

### miR-214 directly targets XBP-1 by interaction with the 3′-UTR

**Figure 2:**
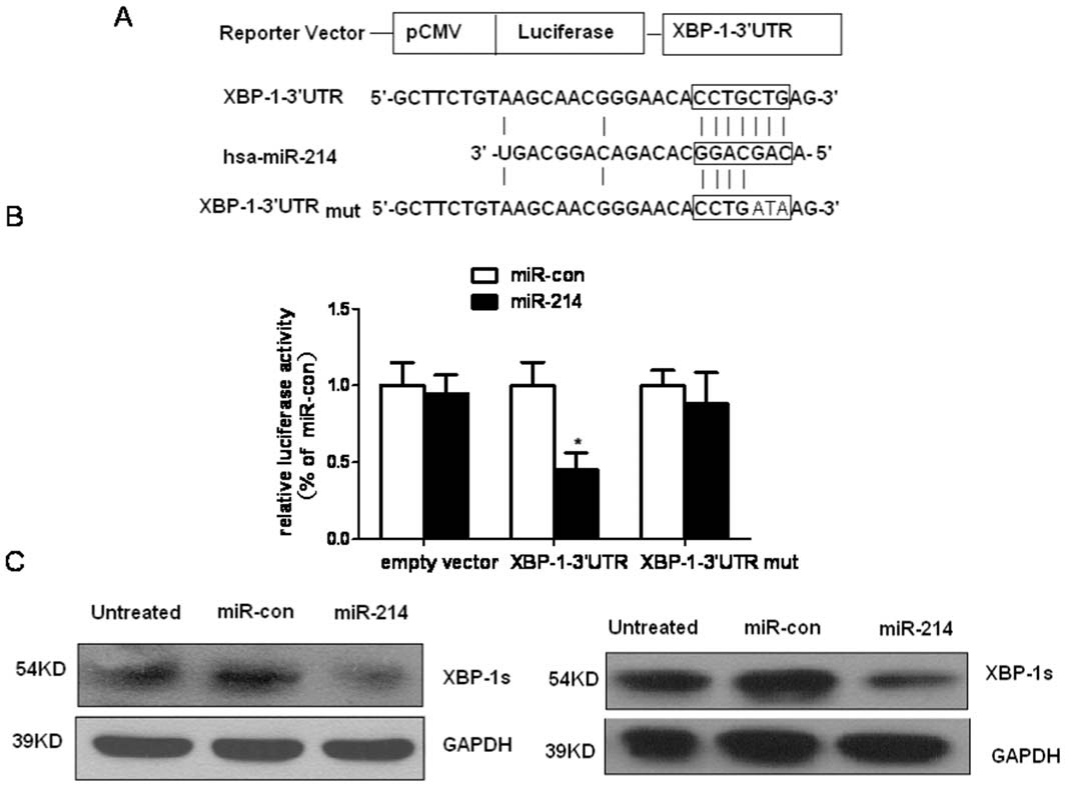
miR-214 negatively regulates XBP-1 through binding to 3′-UTR of the XBP-1. (**A**) Sequence alignment of human miR-214 with 3′-UTR of XBP-1. The seed sequence of miR-214 matches 3′-UTR of XBP-1 for creating the XBP-1 3′-UTR or mutant luciferase reporter construct. (**B**) miR-214 inhibits XBP-1 3′-UTR reporter but not mutant reporter or empty reporter activity. HepG2 cells were transiently transfected with indicated plasmids with miR-control, miR-214 mimics. Following 36 h of incubation, cells were subjected to luciferase assay. Columns, mean of three independent experiments; bars, SD. * P<0.05 vs miR-con. (**C**) miR-214 reduces XBP-1 expression in HepG2 (left) and SMMC-7721(right) cells analyzed by western blotting.

Identification of miRNA-regulated gene targets is a necessary step to understand miRNA functions. Although miR-199a-3p and miR-199a-5p have been reported to contribute to liver carcinogenesis [5], [21], [22], the role of miR-214 in HCC tumorigenesis has not been elucidated. Therefore, we next searched for the target genes of miR-214 in HCC. Putative miR-214 targets were predicted using target prediction programs, miRBase and TargetScan. We found that sequence alignment of hsa-miR-214 with 3′-UTR of the human XBP-1 gene identified a miR-214 binding site (Figure 2A). To confirm that XBP-1 is a putative target of miR-214, we constructed two luciferase reporter vectors with wild-type XBP-1 3′UTR and mutated XBP-1 3′UTR (the complementary sequence in the seed region of miR-214 binding site was mutated). When co-transfected with miR-214 mimics into HepG2 cells, the relative luciferase activity of a XBP-1 3′UTR luciferase reporter was significantly suppressed by ∼50% compared with the transfection of negative control. In contrast, no change in relative luciferase activity was observed in cells transfected with the mutant reporter or empty vector (Figure 2B). These results suggest that miR-214 targets XBP-1 by directly binding the 3′ UTR of XBP-1.

We further found that transient transfection of HepG2 and SMMC-7721 cells with miR-214 efficiently reduced XBP-1 protein levels detected by western blotting analysis (Figure 2C), which was independent of ATF6 and IRE1 signaling (Figure S1). Similar results were attained in human epithelial cervical cancer Hela cells (Figure S2). These data suggest that miR-214 directly recognizes the 3′UTR of XBP-1 mRNA and inhibits XBP-1 translation.

Moreover, western blot analysis showed that XBP-1 protein level was increased in miR-214-downregulated human HCC tissues compared with adjacent nontumorous liver tissues (Figure S3). Our analysis revealed that XBP-1 was a potential target of miR-214.

### miR-214 regulates HCC cell proliferation and apoptosis

**Figure 3:**
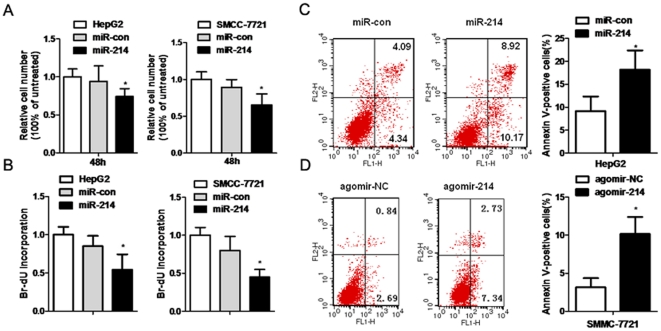
miR-214 regulates HCC cell proliferation and apoptosis. (A) Relative cell number of HepG2 and SMMC-7721 cells at 48 hours post-transfection was evaluated using CCK-8 assay. (**B**) Cell growth rate of HepG2 and SMMC-7721 cells at 48 hours post-transfection was evaluated using Br-dU incorporation assay. (**C**) After 48 hours miR-214 mimics transfection, cells were labeled with Annexin V and analyzed by flow cytometry. (**D**) After 48 hours agomir-214 treatment, SMMC-7721 cells were labeled with Annexin V and analyzed by flow cytometry *P<0.05 vs untreated (HepG2 or SMMC-7721) or miR-con-treated.

Previous studies have implicated XBP-1 as an essential survival factor for ER stress and tumor growth [30], thus we chose whether miR-214 exert an opposite effect in HCC for further investigation. To determine the impact of the miR-214 on HCC cell proliferation, HepG2 and SMMC-7721 cells, were respectively transfected with miR-214 mimics or miR-control and analyzed for cell growth. The CCK-8 proliferation assay showed that cell growth was decreased in miR-214 mimics-transfected HCC cells compared with miR-control -transfected cells or untreated cells (Figure 3A). Similar results were observed by Br-dU incorporation assay in HepG2 and SMMC-7721 cells (Figure 3B). The results of the *in vitro* assays indicated that exogenous miR-214 significantly inhibited the proliferation of hepatoma cell lines.

Further, we tested whether upregulated miR-214 induces HCC cell apoptosis and cell death, by determining the number of early and late apoptotic HepG2 cells following treatments with miR-214 mimics by flow cytometric analysis. As expected, few Annexin V-positive cells were detected in the miR-control-treated or untreated cells, whereas miR-214 restoration increased the percentage of apoptotic cells (∼20% in HepG2) as judged by Annexin V staining (Figure 3C). Consistently, similar effects were also detected in SMMC-7721 cells treated with cholesterol-conjugated 2′-O-methyl-modified miR-214 mimics (agomir-214) (Figure 3D). These results indicate a growth-inhibitory role of miR-214 in HCC.

### miR-214 regulates HCC tumor formation and growth in vitro and in vivo

**Figure 4:**
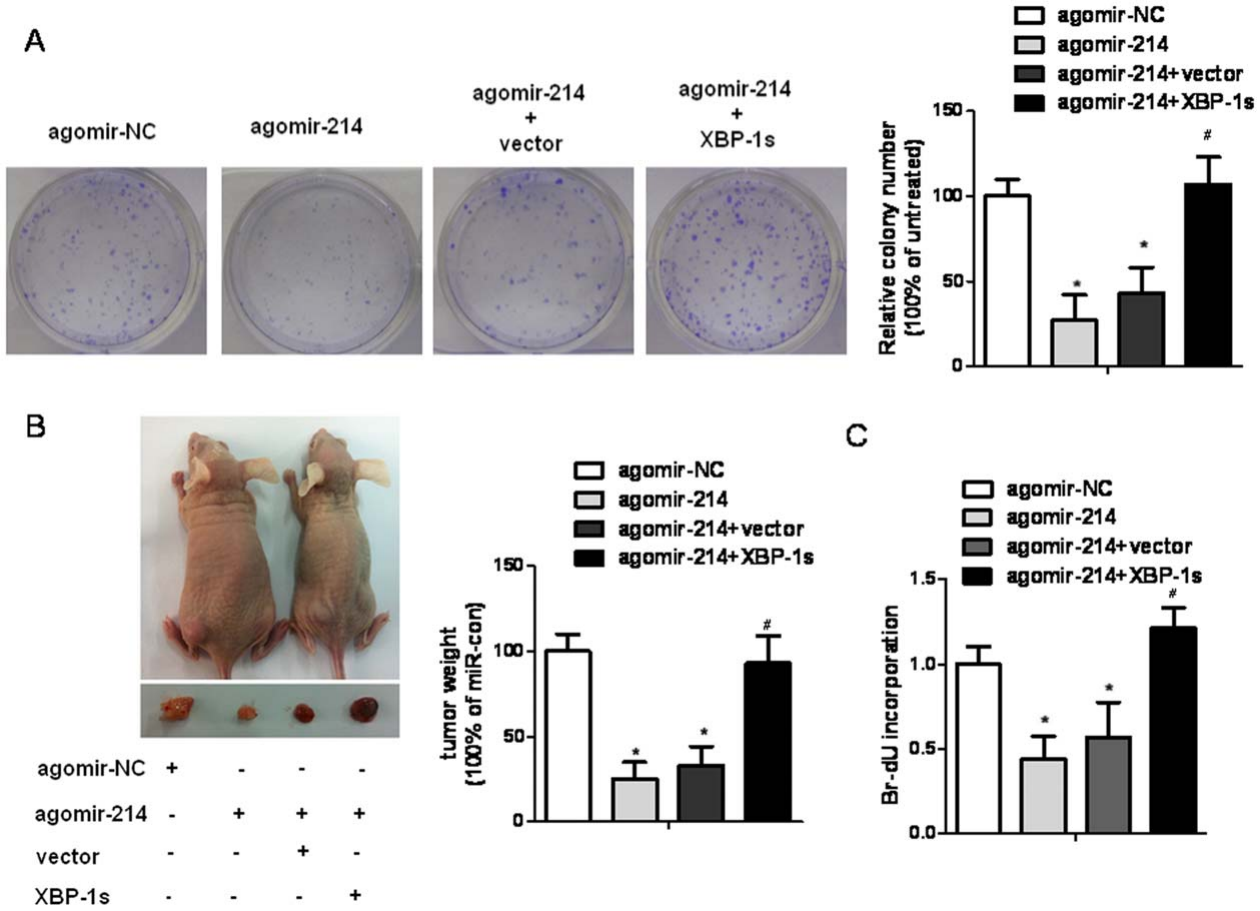
Restored XBP-1 expression reduced miR-214 overexpression induced HCC tumor suppression in vitro and in vivo. (**A**) XBP-1 reintroduced reversed the suppression of colony formation induced by agomir-214 treatment in SMCC-7721 cells. (**B**) XBP-1 reintroduced reversed the suppression of tumor formation induced by agomir-214 treatment in nude mouse SMCC-7721 cells xenograft model. Tumor weight 4 weeks after inoculation was measured. (**C**) XBP-1 reintroduced reversed the suppression of cells proliferation induced by agomir-214 treatment in SMCC-7721 cells. The results were reproducible in three independent experiments. *P<0.05 vs agomir-NC-treated; #P<0.05 vs agomir-214 or agomir-214+vector-treated.

The above findings prompted us to explore the biological significance of miR-214 in HCC tumorigenesis in vivo. As an initial step, the capacity of colony formation was evaluated on SMMC-7721 cells transfected with agomir-214 and agomir negative control (agomir-NC). Results showed that agomir-214-treated cells displayed much fewer and smaller colonies compared with agomir-NC treated cells (Figure 4A), which were consistent with the cell proliferation assay. Further, agomir-NC- and agomir-214-treated SMMC-7721 cells were injected s.c. into either posterior flank of the same nude mice, respectively. After 4 weeks, the size of the tumor nodules was examined. We found that the tumor sizes and tumor weight at the end of observation were significantly decreased in the agomir-214 treatment group compared with that in the agomir-NC treatment group (Figure 4B). Similar results were also found in HepG2 cell xenografts trandfected with miR-214 mimics in vivo (Figure S4 and S5).These results suggest that miR-214 may function as a putative tumor suppressor in HCC cells.

To further verify a potent role for the miR-214/XBP-1 pathway in mediating tumor cells survival and in regulating HCC tumor growth, we re-expressed XBP-1 in miR-214 treated HCC cells. The results showed that restored XBP-1 expression by transfecting pCMV-XL5-XBP-1s attenuated agomir-214 mediated XBP-1 suppression and tumor suppressor effects *in vitro* and *in vivo* (Figure S6 and 4A–C).

### Unfolded protein response downregulates miR-199a/214 expression in HCC cells

**Figure 5:**
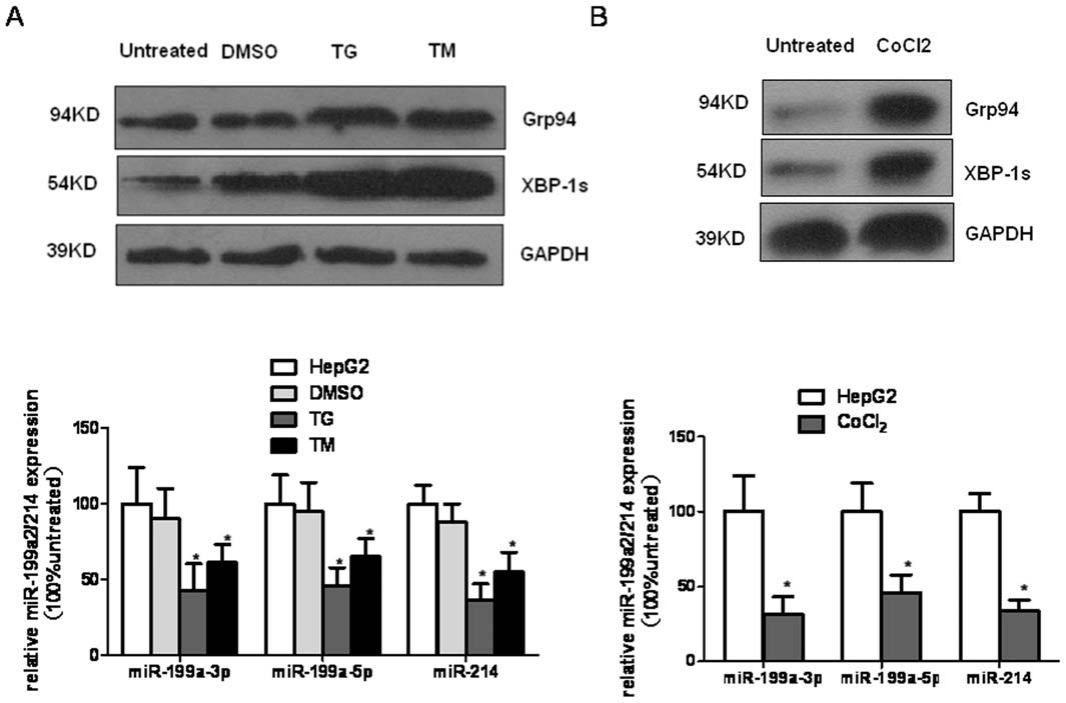
ER stress induces miR-199a/214 downregulation in HCC cells. (**A**) HepG2 cells treated with Thapsigargin(TG, 5 µmol/L) and tunicamycin (TM, 5 µg/ml) for 24 h were analyzed by western blotting for GRP94 and XBP1 expression levels and analyzed by real-time RT-PCR for miR-199a-3p/-5p and miR-214 expression. (**B**) HepG2 cells treated with CoCl_2_ (100 µM) for 24 h were analyzed by Western blotting for GRP94 and XBP1 expression levels and analyzed by real-time RT-PCR for miR-199a-3p/-5p and miR-214 expression. Columns, mean of three independent experiments; bars, SD; *P<0.05 vs untreated (HepG2) or DMSO-treated.

As miR-214 targets XBP-1 and XBP-1 is a key effector of UPR and ER stress [31], [32], we further investigated whether there might be a link between miR-214 down-expression and the activation of UPR in HCC in hepatoma cells. To validate the impact of UPR on miR-214 expression in HCC cells, two classic UPR inducer thapsigargin (TG) and tunicamycin (TM) was used to induce activation of the UPR in HepG2 cells. Incubation of HepG2 cells with TG (5 µmol/L) and TM (5 µg/mL) markedly elevated the GRP94 and XBP1 splicing protein level, indicating UPR activation. The real-time RT-PCR results showed that miR-214 expression was significantly down-regulated in HepG2 cells after TG and TM treatment for 24 h (Figure 5A). As miR-199a-3p, miR-199a-5p and miR-214 sequences were a cluster, we also found that levels of miR-199a-3p and miR-199a-5p were decreased compared with a vehicle group (Figure 5A). In addition, we further tested the effect of hypoxia-induced UPR on the level of miR-199a/214 expression. The expression levels of miR-199a and miR-214 were also lower in HCC cells exposed to anoxia induced by CoCl_2_ (100 µmol/L) (Figure 5B). Thus, miR-199a and miR-214 expression levels are down-regulated by UPR under various physiological and pathological conditions.

### NFκB is a potential negative regulator of the miR-199a-2/miR-214 gene

**Figure 6:**
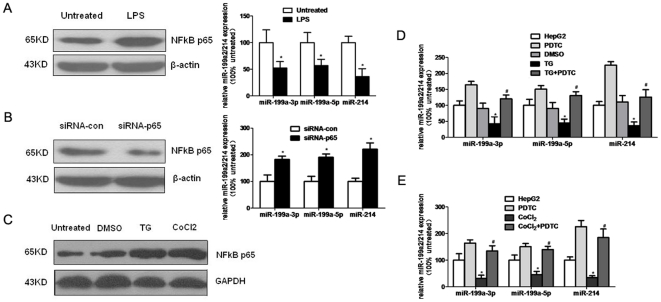
The regulatory role of NFκB in miR-199a/214 Expression. (**A**) LPS (10 µg/ml) treatment induced NFkB p65 expression, attenuates the miR-199a/214 expression in SMMC-7721 cells. (**B**) siRNA specifically targeting human NF-kB P65 subunit (100 nM) dereased NFkB p65 expresssion and increased the expression of miR-199a/214 in SMMC-7721 cells. (**C**) NF-κB is activated by ER Stress and hypoxia in HepG2 cells analyzed by Western blotting. (**D, E**) miR-199a/214 expression in HepG2 cells treated with ER stress inducer (5 µmol/L TG and 100 µM CoCl_2_) and NFκB inhibitors PDTC (100 µM). *P<0.05 vs untreated (HepG2 or SMMC-7721) or DMSO or siRNA con-treated; #P<0.05 vs TG or CoCl_2_ treatment.

As shown in Figure S7, miR-199a2 and miR-214 were regulated as a cluster from pri-miR-199a2 within the human Dnm3os genes; we next examined the miR-199a2 promoter region for transcription factor binding sites, and identified 3 potential putative NFκB binding sites in a 1.2-kb DNA fragment upstream to the pre-miR-199a2 (Figure S8). So we tested whether NFκB is involved in the regulation of miR-199a2/214 expression. As shown in Figure 6A, lipopolysaccharide (LPS) (10 µg/ml), a known inducer of NFκB activity, induced NFκB activation and inhibited miR-199a2/214 expression in SMMC-7721 cells. In contrast, transfection of siRNA targeting human p65 reduced NFκB expression and increased miR-199a2/214 level in SMMC-7721 cells (Figure 6B). These data indicated that NFκB is a potential negative regulator of the miR-199a-2/miR-214 cluster.

Further we found that exposure to TG induced NFκB activation in HepG2 cells (Figure 6C), and suppressed miR-199a2/214 expression (Figure 5A). Consistent with these results, the potent NFκB inhibitor pyrrolidinedithiocarbamate (PDTC) (100 µM) significantly reversed the suppressive effect of UPR on miR-199a2/214 expression (Figure 6D), highlighting the importance of the UPR/NFκB pathway in miR-199a2/214 expression.

Further, we also explored whether NFκB is able to regulate miR-199a/214 expression upon anoxia. Results showed that 100 µmol/L CoCl_2_ treatment also induced NFκB and decreased miR-199a2/214 levels in HepG2 cells simultaneously, and inhibition of NFκB attenuated the reduction of miR-199a2/214 levels observed after anoxic treatment (Figure 6C and E). Thus, these data suggested that UPR mediated the negative regulation of miR-199a2/214 though activating NFκB to participate in HCC progression.

## Discussion

MiRNAs are negative regulators of gene expression and can function as tumor suppressors or oncogenes [33]. Some particular miRNAs were reported to be differentially expressed in different cancer, such as the miR-199a/214 cluster. Increased level of miR-214 was found in ovarian cancer [28], gastric cancer [34] and melanoma [26], inducing chemotherapy resistance or tumor metastasis. But in cervical cancer [27], [29] and breast cancer [35], miR-214 expression was reduced, suggesting a tumor suppressor gene-like function. The possible explanation was that individual miRNAs targets multiple targets in different cells. Several miR-214 targets have been characterized in various tumor types (ovarian cancer, cervical cancer and melanoma) including MEK3, JNK1 [29], PTEN [28], [36], Plexin-B1 [27], Ezh2 [35], [37], and TFAP2C [26] and so on. In our study, we further identified XBP-1 as a new target of miR-214 by binding its 3′-UTR in HCC cells. Moreover, we found that the expressions of Ezh2 and plexin-B1 were not negatively correlated with miR-214 in miR-214-downregulated HCC tumor samples (data not shown). Based on those observations, we assumed that XBP-1, but not Ezh2 and plexin-B1, is the “primary” target of miR214 in HCC, depending on the different cellular context.

As we know, XBP-1, a major transcriptional regulator of the unfolded protein response, regulates a subset of ER resident chaperone genes in the unfolded protein response to protect cancer cells from an inadequate environment such as hypoxia or glucose deprivation [31], which are commonly encountered by most solid tumors including HCC. Clonogenic survival of the XBP-1-deficient tumor cells was significantly reduced during severe hypoxia/anoxia in vitro and the XBP-1-knockout tumor cells were unable to grow as tumors in vivo [30], suggesting that XBP-1 is essential for tumor cell survival under hypoxic conditions and solid tumor formation and growth. Previous studies demonstrated that elevation of the splicing of XBP-1 mRNA, resulting in the activation of XBP-1product, as well as Grp78 and ATF6, occurred in HCC tissues with increased histological grading [38]. Similarly, we found that XBP-1 protein level was increased in miR-214-downexpressed human HCC tissues. Together, these studies indicated that down-regulated miR-214 in HCC cancer induces the over-expression of XBP-1, which in turn accelerates tumorigenesis. Interestingly, similar result of miR-214-mediated XBP-1 repression was also attained in Hela cells. It is consistent with recent reports that miR-214 is down-regulated in human cervical cancer and negatively regulates Hela cell proliferation [27], [29]. It will be interesting now to determine whether over-expression of miR-214 or modulation of its targeting could provide a new treatment modality for miR-214-deficient tumor, such as HCC, cervical cancer and breast cancer.

In the other hand, our study showed that a significant down-regulation of the miR-199a/214 cluster was observed in human HCC tissues and HCC cell lines when compared with normal liver, consistent with previous observations from profiling of miRNAs expression in HCC [9], [39], [40], [41], [42]. But what is the mechanism of the miR-199a/214 cluster down-expression in HCC? Growing evidence has revealed that during ER stress, the UPR represents an adaptive mechanism that supports survival and chemoresistance of tumor cells, and has also been emerging as a means for tumor cells to increase survival under conditions of metabolic stress, hypoxia, and perhaps even chemotherapy [43]. Additionally, activation of MEK/ERK and mTOR have been reported to play a critical role in controlling cell survival or cell death signaling induced by ER stress [44], [45], [46]. As the UPR transcription factor XBP-1 was identified as a target of miR-214 and recent studies have revealed the important functions of miR-199a/b-3p in HCC carcinogenesis and progression by targeting mTOR and c-Met or PAK4/Raf/MEK/ERK Pathway in HCC cells [5], [21], we decided to further investigate the correlation between UPR activation and miR-199a/214 down-expression. Result show that miR-214 and miR-199a-3p/5p was significantly down-regulated in HepG2 cells after TG and TM treatments or anoxia, further suggesting that UPR activated XBP-1 or mTOR and ERK pathway to protect tumor cell survival though suppression of the miR-199a2/214 cluster in HCC.

**Figure 7:**
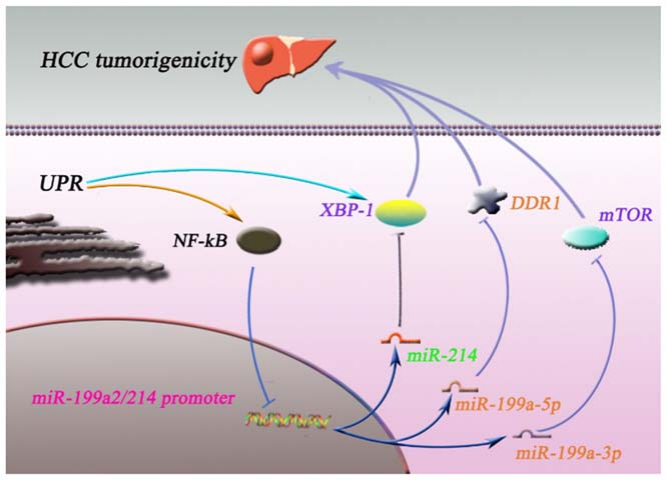
Summary diagram describes the ER stress/NFκB/miR-199a/214 network that regulates HCC tumorigenicity. The miR-214/XBP-1 pathway was shown in this work, while miR-199a-3p/mTOR and miR-199a-5p/DDR1 pathways were reported by other studies.

To start unraveling the regulatory mechanisms of miR-199a2/214 expression under UPR conditions in greater detail, we further found that UPR activated NFκB with concomitant suppression of miR-199a2/214 transcription, and this suppression was reversed by NFκB inhibitor PDTC in HepG2 cells, which suggested that NFκB is a potential negative regulator of the miR-199a-2/miR-214 cluster. NFκB is an important transcription factor that has emerged as an key modulator of altered gene programs and malignant phenotype in development of cancers [47]. Early studies indicated that many cancers, such as breast cancer, lung cancer, and lymphoma, and HCC, constitutively express high levels of NFκB [48], [49]. In addition, hypoxia in HCC cells and tissues induced NFκB overexpression and/or constitutive activation [50], [51]. There was a regulatory Sp1/NFkB/HDAC/miR-29b network to control oncogene expression in acute myeloid leukemia (AML) [52]. In our study, we showed that NFκB and XBP-1 were predominantly expressed but miR-214 was significantly reduced in human HCC tissues, miR-214 directly targets XBP-1, and UPR or hypoxia induced-NFκB activation negatively controls the miR-199a/214 cluster transcription in HCC cells. Therefore, a new UPR/NFκB/miR-214/XBP-1 regulatory circuitry was suggested in HCC progression, in which NFκB was activated by UPR and participated in the negative regulation of miR-199a/214 to regulate HCC progression (Figure 7). This regulatory mechanism partly explained why PDTC treatment inhibited NFκB activation, promoted HCC cells apoptosis and suppressed tumor growth [53], while LPS treatment induced NFκB activation and promoted tumor cell proliferation and metastatic growth [54], [55], [56], [57]. Certainly, more evidences of NFκB bind site in the promoter of the miR-199a/214 cluster are needed in future to support this hypothesis and more investigations are needed to elucidate whether ER stress also activate other factors (e.g.Sp1) together involved in the downregulation of miR-199a/214 in HCC. Nevertheless, further understanding of the molecular mechanism and network by which the miR-199a/214 cluster functions may provide new avenues of research that could aid early diagnosis and treatment of this highly malignant tumor.

In summary, our findings revealed that ER stress suppresses the expression of the miR-199a/214 cluster by activating NFκB to upregulate pro-survival XBP-1 expression, which suggested a novel UPR/NFκB/miR-214/XBP-1 regulatory circuitry whose dysfunction may contribute to tumor survival and progression of HCC. Our study offer new insight into the tumor suppressor activity conferred by miR-199a/214 and the potential mechanisms of hepatocarcinogenesis.

## Materials and Methods

### Materials and reagents

Materials were obtained from the follow suppliers: antibodies against XBP-1, ATF6, NFkB, GAPDH, and β-actin were from santa cruz biotechnology Inc.(Santa cruz, CA); antibodie against GRP94 from Cell Signaling Technology (Beverly, MA); antibody aganist p-IRE1 were from Thermo Scientific Pierce Antibodies (Rockford, IL); cell counting kit-8 were obtained from Beyotime Institute of Biotechnology (Nantong, China); Br-dU cell proliferation ELISA kit was obtained from Roche (Penzberg, Germany); pCMV6-XL5-XBP-1s plasmid was purchased from Origene (Rockville, MD); miRNA negative control (miR-con and anti-miR-con), miR-214 mimics and anti-miR-214, agomir-214 and agomir-NC, antagomir-214 and antagomir-NC, siRNA targeting human NFκB/p65 were obtained from RiboBio (Guangzhou, China); All other chemicals and reagents were purchased from Sigma-Aldrich (Sigma-Aldrich China Inc., Shanghai, China) unless otherwise specified.

### Human tumor samples

A total of 23 snap-frozen normal and malignant liver tissues (14 men and 9 women; median age, 65.0 y; range, 50–78 y) were collected at Tongji hospital (Wuhan, Hubei China). Human normal liver tissues were obtained from distal normal liver tissue of liver hemangioma patients. This study was approved by the Review Board of Tongji Hospital and Tongji Medical College. The subjects recruited to the study provided written informed consent. The investigation conforms to the principles outlined in the Declaration of Helsinki. Tissue samples were obtained and kept frozen in liquid nitrogen and then stored at −80°C until use.

### Cell culture

Human hepatoma cell line HepG2 and human cervical cancer cell line Hela were obtained from the American Type Culture Collection (Manassas, VA), and Human hepatoma cell line SMMC-7721 was from the Committee on Type Culture Collection of Chinese Academy of Sciences (Shanghai, China), and maintained as recommended by the source. Cells were cultured in DMEM, adjusted to contain 4 mM L-glutamine, 1.5 g/l sodium bicarbonate, 4.5 g/l glucose, 10% FBS, 100units/ml penicillin, and 65units/ml streptomycin. All cell lines were maintained at 37°C in a humidified incubator containing 5% CO_2_.

### Cell transfection and treatments

HepG2 and SMMC-7721 cells were transfected with miRNA/siRNA negative control (miR-con and siR-con), miR-214 mimics or siRNA targeting human NFκB/p65 (RiboBio, Guangzhou, China) using Lipofectamine 2000 (Invitrogen, Carlsbad, CA) following the manufacturer's protocol. The siRNA sequences for human NFκB/p65 were: 5′- GCC CUA UCC CUU UAC GUC A-3′ [58]. SMMC-7721 cells were treated with 100 nM agomir-214 and agomir-NC, antagomir-214 and antagomir-NC and were collected 24 hours after treatment for xenotransplantation into nude mice modle and Br-dU incorporation assay. HepG2 Cells were treated with 5 µg/ml of thapsigargin and 5 µmol/L tunicamycin and collected 24 hours after treatment for western blotting (WB) analyses and RNA extraction. Hypoxic conditions were created by using 100 µM of CoCl_2_ for 24 hours before cell collection.

### Western blot analysis

Proteins from cell lysates (20 µg) were separated by 10% SDS-polyacrylamide gel electrophoresis and transferred to a polyvinylidene difluoride membrane. After blocking in 5% nonfat milk, protein blots were incubated with a specific antibody followed by incubation with a peroxidase-conjugated secondary antibody in blocking buffer. The bands were visualized with the enhanced chemiluminescence method according to manufacturer's instructions (Pierce Chemical Co., Rockford, IL).

### RNA extraction and real-time quantitative PCR

Total RNAs (miRNA and mRNA) were extracted using TRIzol (Invitrogen, Carlsbad, CA) according to the manufacture's protocol. Reverse transcription of total miRNA were done starting from equal amounts of total RNA/sample (1 µg) using EasyScript First-Strand cDNA Synthesis SuperMix (TransGen Biotech, Beijing, China). The miR-214, miR-199a-3p and miR-199a-5p level was quantified by real-time quantitative-PCR using TransStartTM SYBR Green qPCR Supermix (TransGen Biotech, Beijing, China), and with U6 small nuclear RNA as an internal normalized reference. The expressions of pre-miR-199a1, pre-miR-199a2, and pre-miR-214 were measured by quantitative RT-PCR, as described previously [59], [60]. The qRT-PCR results were analyzed and expressed as relative miRNA levels of the CT (cycle threshold) value, which was then converted to fold change.

### Vector construction and Luciferase reporter assay

The 3′UTR of the human XBP-1 gene was PCR amplified using the XBP-1 primers: 5′GCG CGA GCT CTT CTC TGT CAG TGG GGA CGT CAT3′ and 3′GCG CAA GCT TAG AAG AAA TCA AAC AAG GAT GCT GC5′, and cloned in between the Hind III and Sac I sites of the pMIR-REPORT miRNA expression reporter vector (Ambion, Inc.) using PCR-generated fragment from genomic DNA. The 3′UTR of the human XBP-1 were mutated using an Easy Mutagenesis System kit (TransGen Biotech,Beijing, China). For luciferase reporter assay, HepG2 cells (1×10^5^) were plated in a 24-well plate and then cotransfected with 400 ng of luciferase reporter vector and 20 ng of pRL-TK, using Lipofectamine 2000 (Invitrogen, Carlsbad, CA) following the manufacturer's protocol. Luciferase activity were detected using the Dual-Luciferase Reporter Assay System (Promega, Madison, WI), as described previously [61]. Transfections were done in duplicates and repeated at least thrice in independent experiments.

### Cell proliferation assay

For Br-dU cell proliferation assay, 24 hours after transfection with miR-214 mimics or treated with agomir-214, HCC cells (HepG2 or SMMC-7721) were dispensed with trypsin and replated in 96-well plates. Six hours later, cells in 96-well plate was labeled with Br-dU for 18 hours. Then the levels of Br-dU incorporation were determined using a Br-dU cell proliferation ELISA kit (Roche, Penzberg, Germany). For CCK-8 proliferation assay, cell proliferation was monitored using Cell Counting Kit-8 (CCK-8) (Beyotime Institute of Biotechnology, China). HepG2 and SMMC-7721 cells (3000 per well) were transfected with miR-214 or miR-control in 96-well culture plates. 48 hours later, CCK-8 reagent was added to each well and incubated at 37°C for 1 h. The number of viable cells was assessed according to the manufacturer's protocol.

### Colony formation assay

Twenty-four hours after transfection, 200 transfected HepG2 cells were placed in a fresh cell plate and maintained in DMEM containing 10% FBS for 2 weeks. SMMC-7721 cells were placed in a fresh cell plate and maintained in DMEM containing 100 nM agomir-214 or agomir-NC, 10% FBS for 2 weeks. Colonies were fixed with methanol and stained with 0.1% crystal violet in 20% methanol for 15 min.

### Flow cytometry analysis

The apoptosis assay was performed on HepG2 cells 48 hours after transfected with either miR-214 mimics or miR-control and SMMC-7721 cells 48 hours after treated with agomir-214 or agomir-NC using the Annexin V-FITC Apoptosis Detection Kit (KeyGen Biotech, Nanjing, China) and analyzed by fluorescence -activated cell sorting (FACS).

### In vivo tumorigenesis assay

miR-214 mimics and miR-con transfected HepG2 cells (1×10^6^), or agomir-214 and agomir-NC treated SMMC-7721 cells (1×10^6^) transfected with pCMV-XL5-XBP-1s plasmid individually, and were suspended in 100 µL PBS and then injected s.c. into either side of the posterior flank of the same female BALB/c athymic nude mouse at 5 to 6 weeks of age, as described previously [6], [16], [20], [62], [63], [64]. Tumor size was measured once every 7 days (beginning 7 days after injection) with microcalipers. We analyzed primary tumor growth by measuring tumor length (L) and width (W), and calculated tumor volume according to V = (L×W^2^)×0.5. At the end of the experiment (4 or 5 weeks after cell injection), mice were sacrificed, and primary tumors were removed and weighed. All animal studies were approved by the Animal Research Committee of Tongji College (Permit Number: S211) and were done according to the guidelines of the NIH.

### Statistical analysis

Continuous variables are expressed as mean values ± SD. One-tailed student's t test was used to compare values of test and control samples. P<0.05 was considered significant.

## Supporting Information

Figure S1 **Western blot showing P-IRE1 and ATF6 protein in HepG2 cells transfected with miR-control and miR-214 mimics, GAPDH as loading controls.**
(TIF)

Figure S2 **Western blot showing XBP-1protein in Hela cells transfected with miR-control and miR-214 mimics, β-actin as loading controls.**
(TIF)

Figure S3 **The NFKB and XBP-1s protein expression in miR-214-underexpressed HCC tissues were analyzed by western blotting.**
(TIF)

Figure S4 **Effect of miR-214 on colony formation of hepatoma cell lines.** HepG2 cells were plated at low density (200 cells/well) after transfection by miR-control, miR-214 mimics. Cells were grown for 14 days, fixed, and stained by 0.1% crystal violet. *P<0.05 vs untreated (HepG2) or miR-con-treated.
(TIF)

Figure S5 **Effect of miR-214 on tumour formation in nude mouse HepG2 xenograft model.** miR-con and miR-214 in Fig. S5 indicate the flanks injected with miR-control-transfected and miR-214 mimics-transfected HepG2 cells, respectively. Photographs illustrate representative features of tumour growth and tumor weight 5 weeks after inoculation. Tumor volume 5 weeks after inoculation was measured. *P<0.05 vs miR-con-treated.
(TIF)

Figure S6 **The XBP-1s protein expression in agomir-214 treated hepatoma-xenograft tissues were analyzed by western blotting.**
(TIF)

Figure S7 **pre-miR-199a1, pre-miR-199a2 and pre-miR-214 expression were detected by qRT-PCR in human normal liver and HepG2 cells.** Result show that pre-miR-199a2 and pre-miR-214 expression were markedly decreased in HepG2 cells, while pre-miR-199a1 expression is not altered compared with that in human normal liver, which suggest that pri-miR-199a2 transcription is mainly suppressed in HCC. Data are shown as mean ± SD (n = 3). * p<0.05 vs human normal liver.
(TIF)

Figure S8 **Schematic diagram showing the location of Sp1 and NFkB binding sites on miR-199a2/214 regulatory region on chromosome 1.**
(TIF)

## References

[1] Bartel DP (2004). MicroRNAs: genomics, biogenesis, mechanism, and function.. Cell.

[2] Nana-Sinkam SP, Croce CM (2011). MicroRNAs as therapeutic targets in cancer.. Transl Res.

[3] Tong AW, Nemunaitis J (2008). Modulation of miRNA activity in human cancer: a new paradigm for cancer gene therapy?. Cancer Gene Ther.

[4] Yeung ML, Jeang KT (2011). MicroRNAs and Cancer Therapeutics.. Pharm Res.

[5] Hou J, Lin L, Zhou W, Wang Z, Ding G (2011). Identification of miRNomes in human liver and hepatocellular carcinoma reveals miR-199a/b-3p as therapeutic target for hepatocellular carcinoma.. Cancer Cell.

[6] Su H, Yang JR, Xu T, Huang J, Xu L (2009). MicroRNA-101, down-regulated in hepatocellular carcinoma, promotes apoptosis and suppresses tumorigenicity.. Cancer Res.

[7] Tsai WC, Hsu PW, Lai TC, Chau GY, Lin CW (2009). MicroRNA-122, a tumor suppressor microRNA that regulates intrahepatic metastasis of hepatocellular carcinoma.. Hepatology.

[8] Zeng C, Wang R, Li D, Lin XJ, Wei QK (2010). A novel GSK-3 beta-C/EBP alpha-miR-122-insulin-like growth factor 1 receptor regulatory circuitry in human hepatocellular carcinoma.. Hepatology.

[9] Gramantieri L, Ferracin M, Fornari F, Veronese A, Sabbioni S (2007). Cyclin G1 is a target of miR-122a, a microRNA frequently down-regulated in human hepatocellular carcinoma.. Cancer Res.

[10] Wu N, Liu X, Xu X, Fan X, Liu M (2011). MicroRNA-373, a new regulator of protein phosphatase 6, functions as an oncogene in hepatocellular carcinoma.. FEBS J.

[11] Gramantieri L, Fornari F, Ferracin M, Veronese A, Sabbioni S (2009). MicroRNA-221 targets Bmf in hepatocellular carcinoma and correlates with tumor multifocality.. Clin Cancer Res.

[12] Pineau P, Volinia S, McJunkin K, Marchio A, Battiston C (2010). miR-221 overexpression contributes to liver tumorigenesis.. Proc Natl Acad Sci U S A.

[13] Wong QW, Ching AK, Chan AW, Choy KW, To KF (2010). MiR-222 overexpression confers cell migratory advantages in hepatocellular carcinoma through enhancing AKT signaling.. Clin Cancer Res.

[14] Xu T, Zhu Y, Xiong Y, Ge YY, Yun JP (2009). MicroRNA-195 suppresses tumorigenicity and regulates G1/S transition of human hepatocellular carcinoma cells.. Hepatology.

[15] Yao J, Liang L, Huang S, Ding J, Tan N (2010). MicroRNA-30d promotes tumor invasion and metastasis by targeting Galphai2 in hepatocellular carcinoma.. Hepatology.

[16] Liang L, Wong CM, Ying Q, Fan DN, Huang S (2010). MicroRNA-125b suppressesed human liver cancer cell proliferation and metastasis by directly targeting oncogene LIN28B2.. Hepatology.

[17] Liu WH, Yeh SH, Lu CC, Yu SL, Chen HY (2009). MicroRNA-18a prevents estrogen receptor-alpha expression, promoting proliferation of hepatocellular carcinoma cells.. Gastroenterology.

[18] Wong CC, Wong CM, Tung EK, Au SL, Lee JM (2011). The microRNA miR-139 suppresses metastasis and progression of hepatocellular carcinoma by down-regulating Rho-kinase 2.. Gastroenterology.

[19] Wong QW, Lung RW, Law PT, Lai PB, Chan KY (2008). MicroRNA-223 is commonly repressed in hepatocellular carcinoma and potentiates expression of Stathmin1.. Gastroenterology.

[20] Xiong Y, Fang JH, Yun JP, Yang J, Zhang Y (2010). Effects of microRNA-29 on apoptosis, tumorigenicity, and prognosis of hepatocellular carcinoma.. Hepatology.

[21] Fornari F, Milazzo M, Chieco P, Negrini M, Calin GA (2010). MiR-199a-3p regulates mTOR and c-Met to influence the doxorubicin sensitivity of human hepatocarcinoma cells.. Cancer Res.

[22] Shen Q, Cicinnati VR, Zhang X, Iacob S, Weber F (2010). Role of microRNA-199a-5p and discoidin domain receptor 1 in human hepatocellular carcinoma invasion.. Mol Cancer.

[23] Lee YB, Bantounas I, Lee DY, Phylactou L, Caldwell MA (2009). Twist-1 regulates the miR-199a/214 cluster during development.. Nucleic Acids Res.

[24] Yin G, Chen R, Alvero AB, Fu HH, Holmberg J (2010). TWISTing stemness, inflammation and proliferation of epithelial ovarian cancer cells through MIR199A2/214.. Oncogene.

[25] Sakurai K, Furukawa C, Haraguchi T, Inada K, Shiogama K (2011). MicroRNAs miR-199a-5p and -3p target the Brm subunit of SWI/SNF to generate a double-negative feedback loop in a variety of human cancers.. Cancer Res.

[26] Penna E, Orso F, Cimino D, Tenaglia E, Lembo A (2011). microRNA-214 contributes to melanoma tumour progression through suppression of TFAP2C.. EMBO J.

[27] Qiang R, Wang F, Shi LY, Liu M, Chen S (2011). Plexin-B1 is a target of miR-214 in cervical cancer and promotes the growth and invasion of HeLa cells.. Int J Biochem Cell Biol.

[28] Yang H, Kong W, He L, Zhao JJ, O'Donnell JD (2008). MicroRNA expression profiling in human ovarian cancer: miR-214 induces cell survival and cisplatin resistance by targeting PTEN.. Cancer Res.

[29] Yang Z, Chen S, Luan X, Li Y, Liu M (2009). MicroRNA-214 is aberrantly expressed in cervical cancers and inhibits the growth of HeLa cells.. IUBMB Life.

[30] Romero-Ramirez L, Cao H, Nelson D, Hammond E, Lee AH (2004). XBP1 is essential for survival under hypoxic conditions and is required for tumor growth.. Cancer Res.

[31] Lee AH, Iwakoshi NN, Glimcher LH (2003). XBP-1 regulates a subset of endoplasmic reticulum resident chaperone genes in the unfolded protein response.. Mol Cell Biol.

[32] Koong AC, Chauhan V, Romero-Ramirez L (2006). Targeting XBP-1 as a novel anti-cancer strategy.. Cancer Biol Ther.

[33] Winter J, Jung S, Keller S, Gregory RI, Diederichs S (2009). Many roads to maturity: microRNA biogenesis pathways and their regulation.. Nat Cell Biol.

[34] Ueda T, Volinia S, Okumura H, Shimizu M, Taccioli C (2010). Relation between microRNA expression and progression and prognosis of gastric cancer: a microRNA expression analysis.. Lancet Oncol.

[35] Derfoul A, Juan A, Difilippantonio MJ, Palanisamy N, Ried T (2011). Decreased MicroRNA-214 Levels In Breast Cancer Cells Coincides with Increased Cell Proliferation, Invasion, and Accumulation of the Polycomb Ezh2 Methyltransferase.. Carcinogenesis.

[36] Jindra PT, Bagley J, Godwin JG, Iacomini J (2010). Costimulation-dependent expression of microRNA-214 increases the ability of T cells to proliferate by targeting Pten.. J Immunol.

[37] Juan AH, Kumar RM, Marx JG, Young RA, Sartorelli V (2009). Mir-214-dependent regulation of the polycomb protein Ezh2 in skeletal muscle and embryonic stem cells.. Mol Cell.

[38] Shuda M, Kondoh N, Imazeki N, Tanaka K, Okada T (2003). Activation of the ATF6, XBP1 and grp78 genes in human hepatocellular carcinoma: a possible involvement of the ER stress pathway in hepatocarcinogenesis.. J Hepatol.

[39] Wong CC, Wong CM, Tung EK, Au SL, Lee JM (2011). The microRNA miR-139 suppresses metastasis and progression of hepatocellular carcinoma by down-regulating Rho-kinase 2.. Gastroenterology.

[40] Wong QW, Ching AK, Chan AW, Choy KW, To KF (2010). MiR-222 overexpression confers cell migratory advantages in hepatocellular carcinoma through enhancing AKT signaling.. Clin Cancer Res.

[41] Jiang J, Gusev Y, Aderca I, Mettler TA, Nagorney DM (2008). Association of MicroRNA expression in hepatocellular carcinomas with hepatitis infection, cirrhosis, and patient survival.. Clin Cancer Res.

[42] Wang Y, Lee AT, Ma JZ, Wang J, Ren J (2008). Profiling microRNA expression in hepatocellular carcinoma reveals microRNA-224 up-regulation and apoptosis inhibitor-5 as a microRNA-224-specific target.. J Biol Chem.

[43] Healy SJ, Gorman AM, Mousavi-Shafaei P, Gupta S, Samali A (2009). Targeting the endoplasmic reticulum-stress response as an anticancer strategy.. Eur J Pharmacol.

[44] Qin L, Wang Z, Tao L, Wang Y (2010). ER stress negatively regulates AKT/TSC/mTOR pathway to enhance autophagy.. Autophagy.

[45] Wouters BG, Koritzinsky M (2008). Hypoxia signalling through mTOR and the unfolded protein response in cancer.. Nat Rev Cancer.

[46] Hu P, Han Z, Couvillon AD, Exton JH (2004). Critical role of endogenous Akt/IAPs and MEK1/ERK pathways in counteracting endoplasmic reticulum stress-induced cell death.. J Biol Chem.

[47] Van Waes C (2007). Nuclear factor-kappaB in development, prevention, and therapy of cancer.. Clin Cancer Res.

[48] Qiao L, Zhang H, Yu J, Francisco R, Dent P (2006). Constitutive activation of NF-kappaB in human hepatocellular carcinoma: evidence of a cytoprotective role.. Hum Gene Ther.

[49] Song L, Xiong H, Li J, Liao W, Wang L (2011). Sphingosine kinase-1 enhances resistance to apoptosis through activation of PI3K/Akt/NF-kappaB pathway in human non-small cell lung cancer.. Clin Cancer Res.

[50] Culver C, Sundqvist A, Mudie S, Melvin A, Xirodimas D (2010). Mechanism of hypoxia-induced NF-kappaB.. Mol Cell Biol.

[51] van Uden P, Kenneth NS, Rocha S (2008). Regulation of hypoxia-inducible factor-1alpha by NF-kappaB.. Biochem J.

[52] Liu S, Wu LC, Pang J, Santhanam R, Schwind S (2010). Sp1/NFkappaB/HDAC/miR-29b regulatory network in KIT-driven myeloid leukemia.. Cancer Cell.

[53] Liu LP, Liang HF, Chen XP, Zhang WG, Yang SL (2010). The role of NF-kappaB in Hepatitis b virus X protein-mediated upregulation of VEGF and MMPs.. Cancer Invest.

[54] Liu X, Liang J, Li G (2010). Lipopolysaccharide promotes adhesion and invasion of hepatoma cell lines HepG2 and HepG2.2.15.. Mol Biol Rep.

[55] Harmey JH, Bucana CD, Lu W, Byrne AM, McDonnell S (2002). Lipopolysaccharide-induced metastatic growth is associated with increased angiogenesis, vascular permeability and tumor cell invasion.. Int J Cancer.

[56] Chen R, Alvero AB, Silasi DA, Kelly MG, Fest S (2008). Regulation of IKKbeta by miR-199a affects NF-kappaB activity in ovarian cancer cells.. Oncogene.

[57] Chen R, Alvero AB, Silasi DA, Steffensen KD, Mor G (2008). Cancers take their Toll–the function and regulation of Toll-like receptors in cancer cells.. Oncogene.

[58] Guo J, Verma UN, Gaynor RB, Frenkel EP, Becerra CR (2004). Enhanced chemosensitivity to irinotecan by RNA interference-mediated down-regulation of the nuclear factor-kappaB p65 subunit.. Clin Cancer Res.

[59] Git A, Spiteri I, Blenkiron C, Dunning MJ, Pole JC (2008). PMC42, a breast progenitor cancer cell line, has normal-like mRNA and microRNA transcriptomes.. Breast Cancer Res.

[60] Zhou J, Wang KC, Wu W, Subramaniam S, Shyy JY (2011). MicroRNA-21 targets peroxisome proliferators-activated receptor-{alpha} in an autoregulatory loop to modulate flow-induced endothelial inflammation.. Proc Natl Acad Sci U S A.

[61] Ding H, Wu B, Wang H, Lu Z, Yan J (2010). A novel loss-of-function DDAH1 promoter polymorphism is associated with increased susceptibility to thrombosis stroke and coronary heart disease.. Circ Res.

[62] Jiang S, Zhang HW, Lu MH, He XH, Li Y (2010). MicroRNA-155 functions as an OncomiR in breast cancer by targeting the suppressor of cytokine signaling 1 gene.. Cancer Res.

[63] Veronese A, Lupini L, Consiglio J, Visone R, Ferracin M (2010). Oncogenic role of miR-483-3p at the IGF2/483 locus.. Cancer Res.

[64] Zhang S, Shan C, Kong G, Du Y, Ye L (2011). MicroRNA-520e suppresses growth of hepatoma cells by targeting the NF-kappaB-inducing kinase (NIK).. Oncogene.

