# Supplementary figures and images for: ER Stress Negatively Modulates the Expression of the miR-199a/214 Cluster to Regulates Tumor Survival and Progression in Human Hepatocellular Cancer
Source: PLoS One. 2012 Feb 16;7(2):e31518. doi: 10.1371/journal.pone.0031518 (PMC3281082; doi:10.1371/journal.pone.0031518)

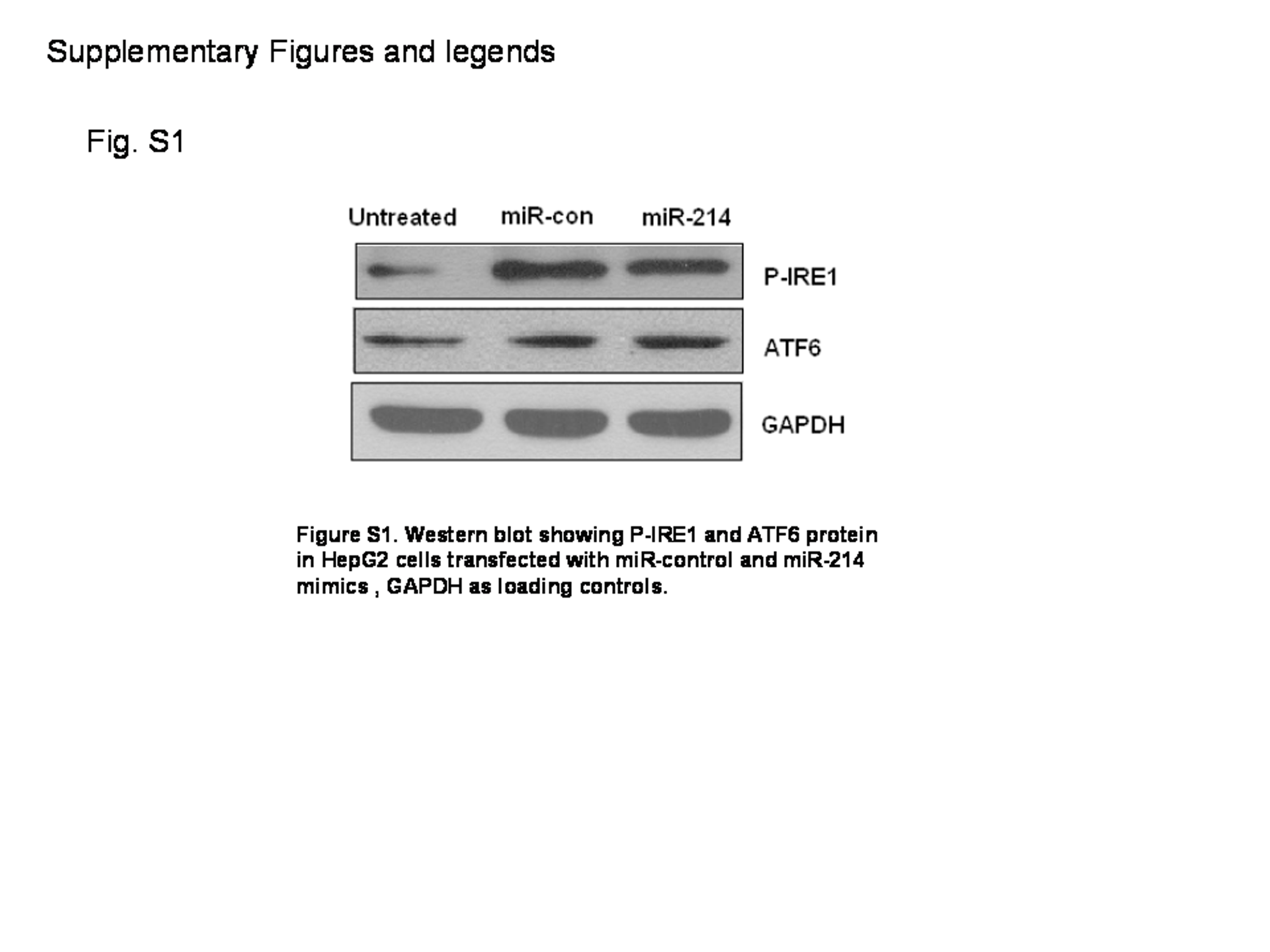

Supplement: Figure S1 — Western blot showing P-IRE1 and ATF6 protein in HepG2 cells transfected with miR-control and miR-214 mimics, GAPDH as loading controls. (TIF) [file pone.0031518.s001.tif]

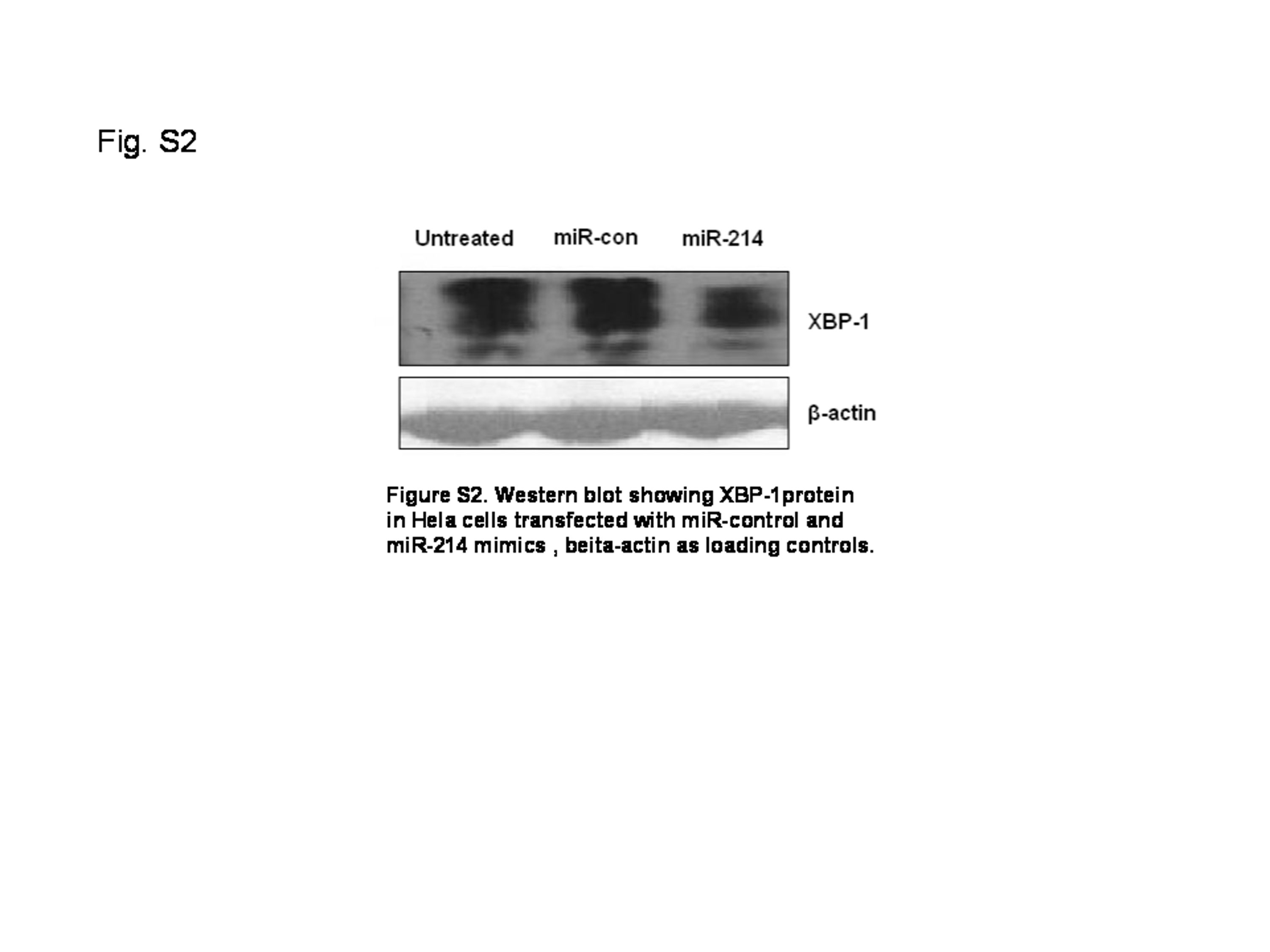

Supplement: Figure S2 — Western blot showing XBP-1protein in Hela cells transfected with miR-control and miR-214 mimics, β-actin as loading controls. (TIF) [file pone.0031518.s002.tif]

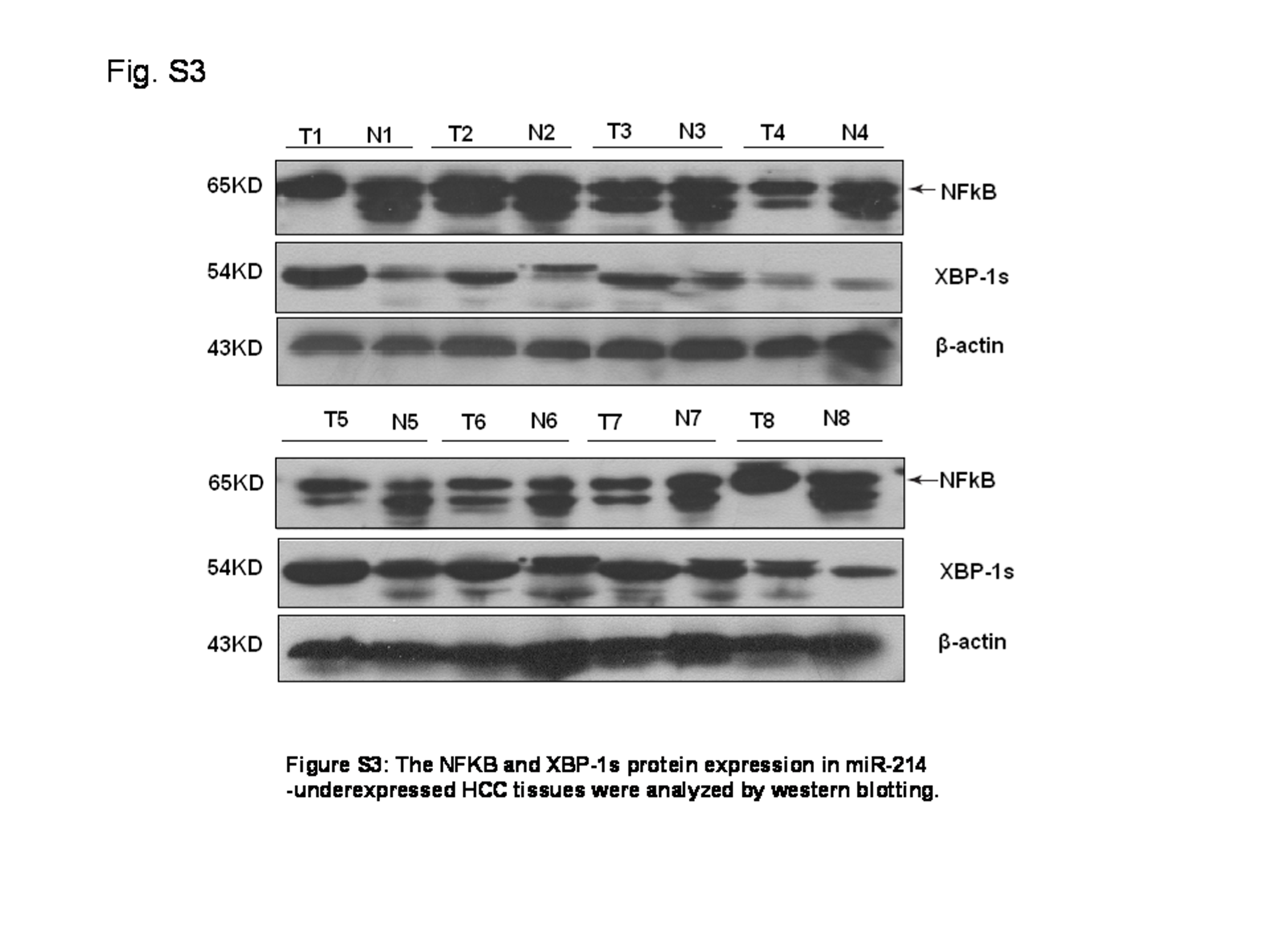

Supplement: Figure S3 — The NFKB and XBP-1s protein expression in miR-214-underexpressed HCC tissues were analyzed by western blotting. (TIF) [file pone.0031518.s003.tif]

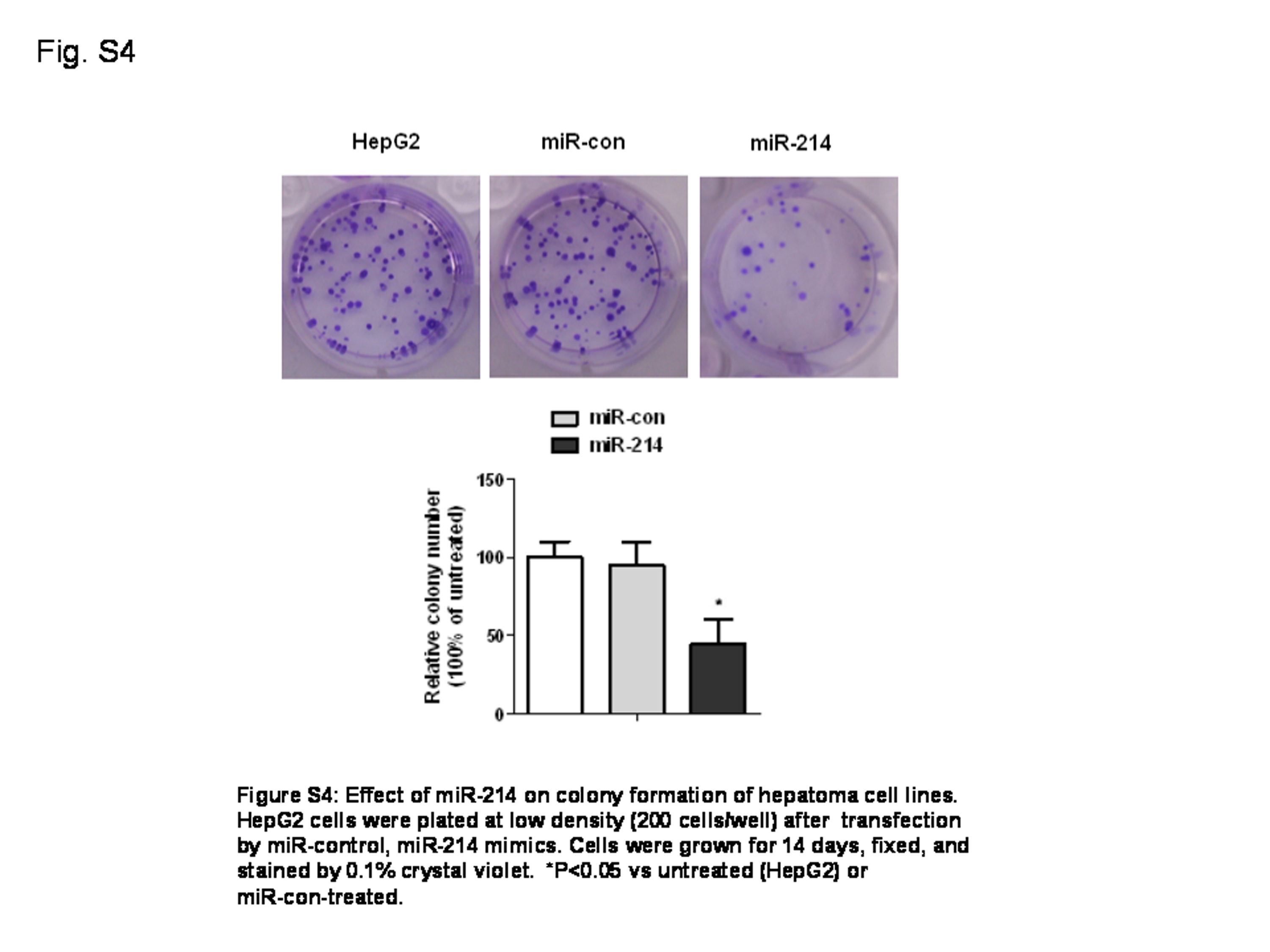

Supplement: Figure S4 — Effect of miR-214 on colony formation of hepatoma cell lines. HepG2 cells were plated at low density (200 cells/well) after transfection by miR-control, miR-214 mimics. Cells were grown for 14 days, fixed, and stained by 0.1% crystal violet. *P<0.05 vs untreated (HepG2) or miR-con-treated. (TIF) [file pone.0031518.s004.tif]

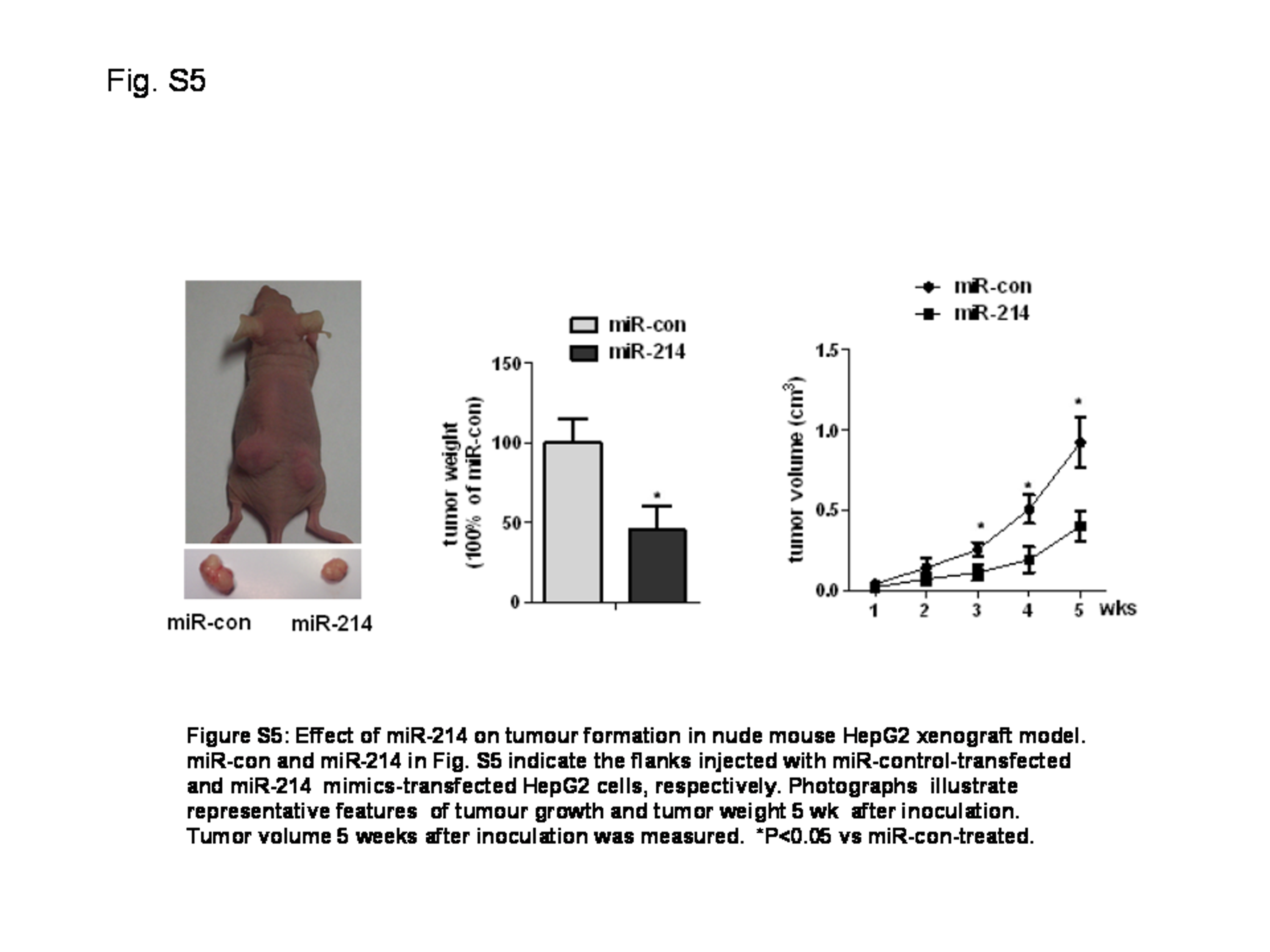

Supplement: Figure S5 — Effect of miR-214 on tumour formation in nude mouse HepG2 xenograft model. miR-con and miR-214 in Fig. S5 indicate the flanks injected with miR-control-transfected and miR-214 mimics-transfected HepG2 cells, respectively. Photographs illustrate representative features of tumour growth and tumor weight 5 weeks after inoculation. Tumor volume 5 weeks after inoculation was measured. *P<0.05 vs miR-con-treated. (TIF) [file pone.0031518.s005.tif]

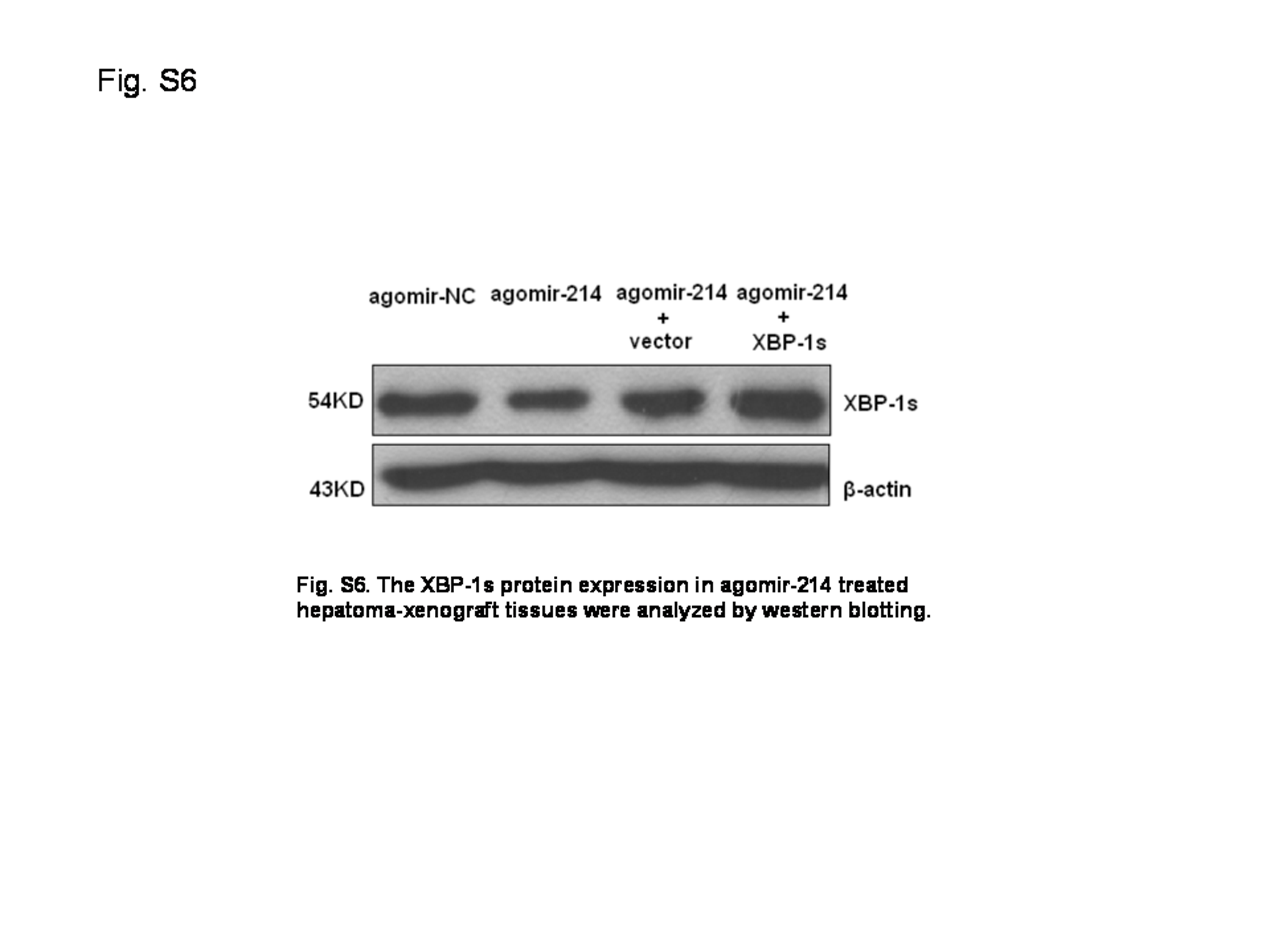

Supplement: Figure S6 — The XBP-1s protein expression in agomir-214 treated hepatoma-xenograft tissues were analyzed by western blotting. (TIF) [file pone.0031518.s006.tif]

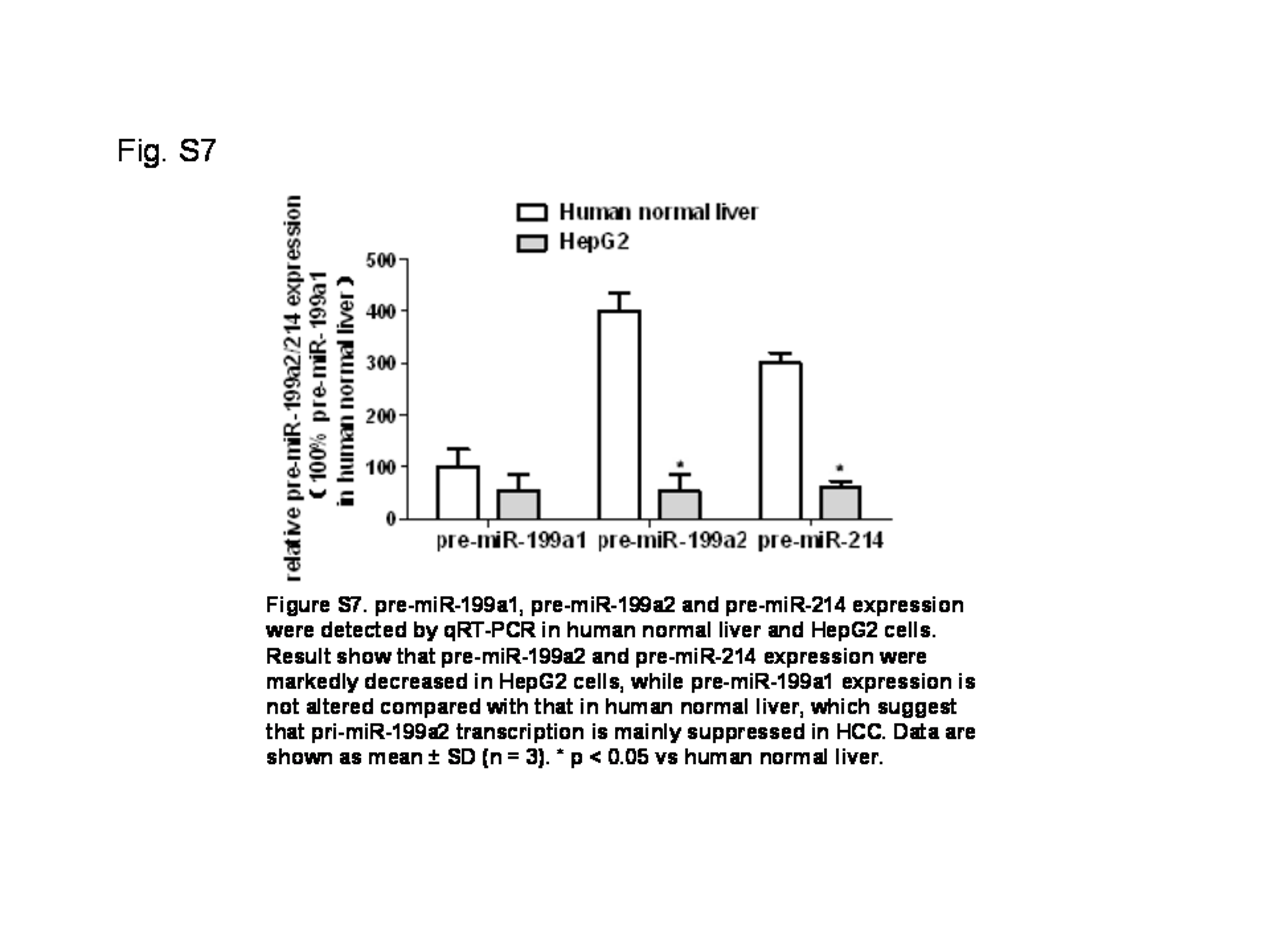

Supplement: Figure S7 — pre-miR-199a1, pre-miR-199a2 and pre-miR-214 expression were detected by qRT-PCR in human normal liver and HepG2 cells. Result show that pre-miR-199a2 and pre-miR-214 expression were markedly decreased in HepG2 cells, while pre-miR-199a1 expression is not altered compared with that in human normal liver, which suggest that pri-miR-199a2 transcription is mainly suppressed in HCC. Data are shown as mean ± SD (n = 3). * p<0.05 vs human normal liver. (TIF) [file pone.0031518.s007.tif]

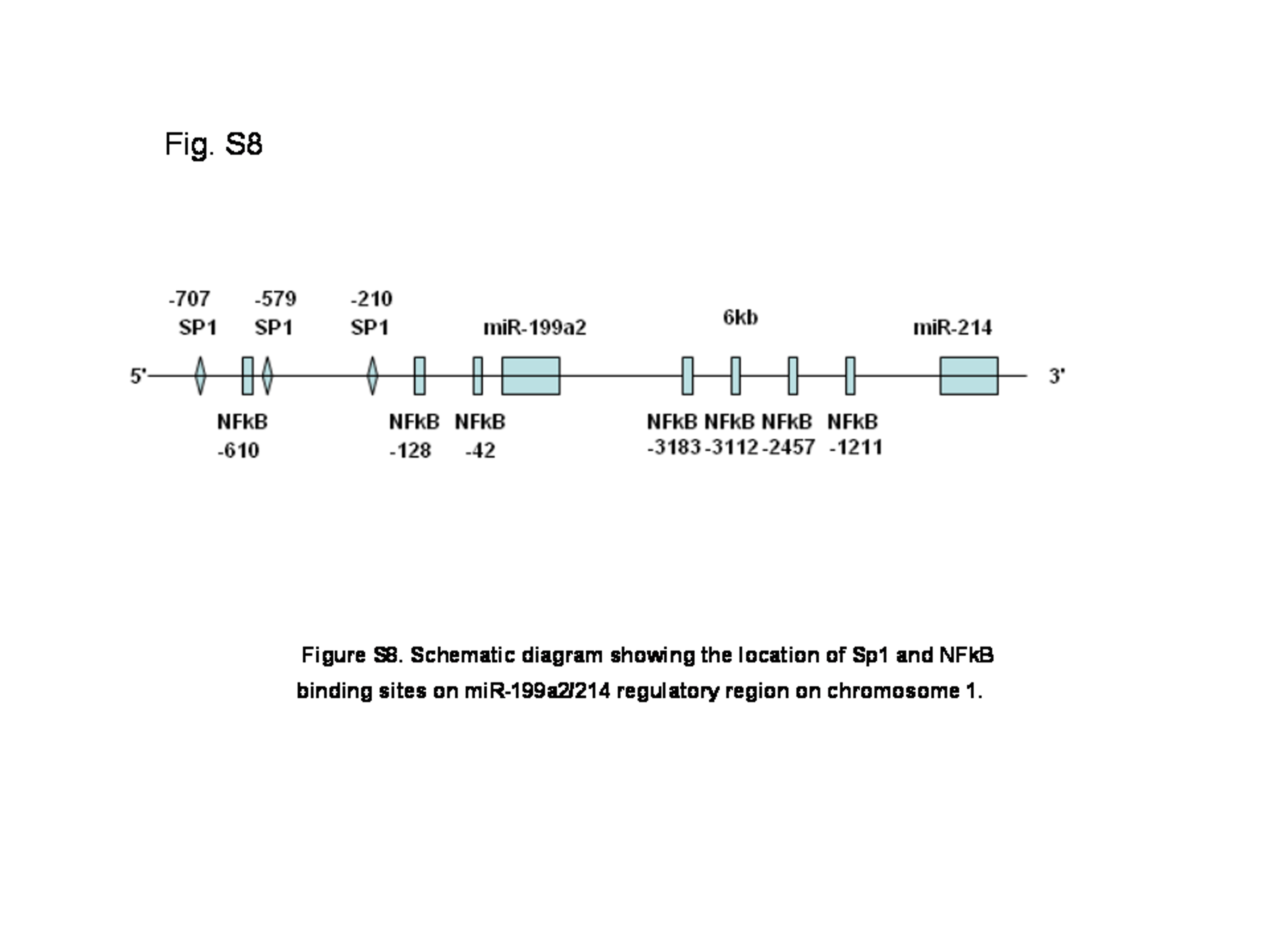

Supplement: Figure S8 — Schematic diagram showing the location of Sp1 and NFkB binding sites on miR-199a2/214 regulatory region on chromosome 1. (TIF) [file pone.0031518.s008.tif]
